# Evolutionary Dynamics of Oropouche Virus in South America

**DOI:** 10.1128/JVI.01127-19

**Published:** 2020-02-14

**Authors:** Bernardo Gutierrez, Emma L. Wise, Steven T. Pullan, Christopher H. Logue, Thomas A. Bowden, Marina Escalera-Zamudio, Gabriel Trueba, Marcio R. T. Nunes, Nuno R. Faria, Oliver G. Pybus

**Affiliations:** a Department of Zoology, University of Oxford, Oxford, United Kingdom; b School of Biological and Environmental Sciences, Universidad San Francisco de Quito USFQ, Quito, Ecuador; c University of Plymouth, Plymouth, United Kingdom; d National Infection Service, Public Health England, Porton Down, United Kingdom; e Microbiology Institute, Universidad San Francisco de Quito USFQ, Quito, Ecuador; f Division of Structural Biology, Wellcome Centre for Human Genetics, University of Oxford, Oxford, United Kingdom; g Center for Technological Innovations, Evandro Chagas Institute, Ministry of Health, Ananindeua, Pará, Brazil; h Center for Biodefense and Emerging Infectious Diseases, Department of Pathology, University of Texas Medical Branch, Galveston, Texas, USA; Cornell University

**Keywords:** Oropouche virus, arbovirus, bunyavirus, emerging infectious diseases, evolutionary biology, phylogenetic analysis

## Abstract

The Amazon basin is home to numerous arthropod-borne viral pathogens that cause febrile disease in humans. Among these, *Oropouche orthobunyavirus* (OROV) is a relatively understudied member of the genus *Orthobunyavirus*, family *Peribunyaviridae*, that causes periodic outbreaks in human populations in Brazil and other South American countries. Although several studies have described the genetic diversity of the virus, the evolutionary processes that shape the OROV genome remain poorly understood. Here, we present a comprehensive study of the genomic dynamics of OROV that encompasses phylogenetic analysis, evolutionary rate estimates, inference of natural selective pressures, recombination and reassortment, and structural analysis of OROV variants. Our study includes all available published sequences, as well as a set of new OROV genome sequences obtained from patients in Ecuador, representing the first set of genomes from this country. Our results show differing evolutionary processes on the three segments that comprise the viral genome. We infer differing times of the most recent common ancestors of the genome segments and propose that this can be explained by cryptic reassortment. We also present the discovery of previously unobserved putative N-linked glycosylation sites, as well as codons that evolve under positive selection on the viral surface proteins, and discuss the potential role of these features in the evolution of OROV through a combined phylogenetic and structural approach.

**IMPORTANCE** The emergence and reemergence of pathogens such as Zika virus, chikungunya virus, and yellow fever virus have drawn attention toward other cocirculating arboviruses in South America. Oropouche virus (OROV) is a poorly studied pathogen responsible for over a dozen outbreaks since the early 1960s and represents a public health burden to countries such as Brazil, Panama, and Peru. OROV is likely underreported since its symptomatology can be easily confounded with other febrile illnesses (e.g., dengue fever and leptospirosis) and point-of-care testing for the virus is still uncommon. With limited data, there is a need to optimize the information currently available. Analysis of OROV genomes can help us understand how the virus circulates in nature and can reveal the evolutionary forces that shape the genetic diversity of the virus, which has implications for molecular diagnostics and the design of potential vaccines.

## Abstract (precis)

The emergence and reemergence of pathogens such as Zika virus, chikungunya virus, and yellow fever virus have drawn attention toward other cocirculating arboviruses in South America. Oropouche virus (OROV) is a poorly studied pathogen responsible for over a dozen outbreaks since the early 1960s and represents a public health burden to countries such as Brazil, Panama, and Peru. OROV is likely underreported since its symptomatology can be easily confounded with other febrile illnesses (e.g., dengue fever and leptospirosis) and point-of-care testing for the virus is still uncommon. With limited data, there is a need to optimize the information currently available. Analysis of OROV genomes can help us understand how the virus circulates in nature and can reveal the evolutionary forces that shape the genetic diversity of the virus, which has implications for molecular diagnostics and the design of potential vaccines.

## INTRODUCTION

The *Bunyavirales* is a highly diverse order of viruses that include multiple emerging human pathogens. Within this order, the *Orthobunyavirus* genus (family *Peribunyaviridae*) includes many arthropod-borne virus species that have been associated with disease in humans (1). The severity of disease varies and ranges from self-limiting febrile illness to encephalitis and hemorrhagic fever (2). One prominent member of this genus is Oropouche virus (OROV), a common causal agent of febrile disease in the Amazon basin (3) and a potential candidate for future emerging epidemics in the region and elsewhere (4).

Since its first description in 1961 in Trinidad and Tobago (5), OROV has caused several outbreaks and sporadic infections in the Brazilian Amazon, particularly in the states of Pará, Amapá, Rondônia, Maranhão, Acre, Amazonas, Minas Gerais, Mato Grosso, and Tocantins (6–9), and evidence suggests the circulation of OROV in other Brazilian states (10). Since the late 1980s, additional cases and outbreaks of OROV have been reported in Panama, Peru (11–13), and, more recently, Ecuador (14). The virus has been isolated from sloths (*Bradypus trydactilus*) (3) and marmosets (*Callithrix* sp.) (15), as well as from invertebrates such as *Culex pipiens quinquefasciatus* (9) and *Ochlerotatus serratus* mosquitoes and *Culicoides paraensis* biting midges (3). It has been proposed that OROV has two distinct transmission cycles (16). In the sylvatic transmission cycle, wild mammals such as sloths (*B. trydactilus*) and primates (*Allouatta caraya*, *Callithrix penicillata*) serve as hosts for the virus, which is transmitted by vector species that are common in rural areas (*Aedes serratus* and *Coquillettidia venezuelensis* have been proposed as vectors in the sylvatic cycle). This sylvatic cycle may possibly include other vertebrate hosts, including wild birds and rodents (such as *Proechimys* sp.). In contrast, the urban transmission cycle involves human-to-human transmission mediated by the biting midge *C. paraensis* (16) and can be facilitated by anthropogenic disturbance of forest areas. OROV produces an acute febrile illness in humans, characterized by unspecific symptoms such as fever, chills, headaches, myalgia, and joint pain (17) and is therefore difficult to diagnose and differentiate from other co-occurring infectious diseases such as leptospirosis, dengue fever, Venezuelan equine encephalitis, malaria, and rickettsial and *Coxiella* infections (18). Specific to the Amazon region, OROV infections are difficult to distinguish from undifferentiated febrile illness associated with group C orthobunyaviruses (e.g., Itaqui and Caraparú viruses) or phleboviruses from the Candiru complex.

The genome of OROV is typical of the orthobunyaviruses and comprises three negative-sense RNA segments of different sizes: a large segment (L) that encodes a RNA dependent RNA polymerase, a medium (M) segment that encodes a membrane polyprotein comprised of three main components (the Gn, NSm, and Gc proteins), and a small (S) segment that encodes the structural nucleocapsid protein N and a second nonstructural protein, Ns, in an overlapping reading frame (2). The Gn and Gc proteins determine the architecture of the viral particle; cryo-electron microscopy studies show that the membrane proteins of Bunyamwera virus (BUNV) form a tripodal structure (19), and the crystal structures of the N terminus of Gc suggest that this trimeric assembly may be conserved across various orthobunyaviruses (20). The segmented nature of the OROV genome enhances the likelihood of genome reassortment events, a phenomenon that has been observed more generally in the bunyaviruses (2) and has been associated, in some cases, with a dramatic increase in disease severity, such as with Ngari virus, a reassortant between BUNV and Batai virus (21). Furthermore, reassortment appears to play an important role in the evolution and emergence of new viruses within OROV and related species, as has been described for the reassortant Jatobal virus (22) and Perdoes virus (7) in Brazil, the Iquitos virus (IQTV) in Peru (23), and the Madre de Dios virus in Venezuela (24).

Despite a heightened interest in OROV, reflected by an increasing number of publications covering the virus in recent years (16), many aspects of OROV history, biology, and ecology remain poorly understood. Although the evolution of the virus has been previously studied using standard phylogenetic methods (3, 7, 8, 25, 26), a comprehensive analysis of the molecular evolution of OROV that incorporates the complete genetic diversity of the virus (represented by whole genomes) and a comparison of the evolutionary histories of its genome segments has not yet been undertaken. Here, we present the results of an in-depth analysis of the OROV genomes from the Americas that includes the genomes of six new OROV isolates sampled in Ecuador (27) and explores the differences between the evolutionary histories of the three viral segments through comparative phylogenetics. We evaluate the temporal signal of each RNA segment, estimate their dates of origin and rates of evolution, and test for recombination and reassortment. Furthermore, we combine phylogenetic tests for natural selection on the viral genome with structural mapping and N-linked glycosylation sequon analysis to determine the factors that drive the evolution of the viral genome and explain the observed differences between segments.

(This article was submitted to an online preprint archive [28].)

## RESULTS

### Molecular phylogenetics and molecular clock analyses.

**FIG 1:**
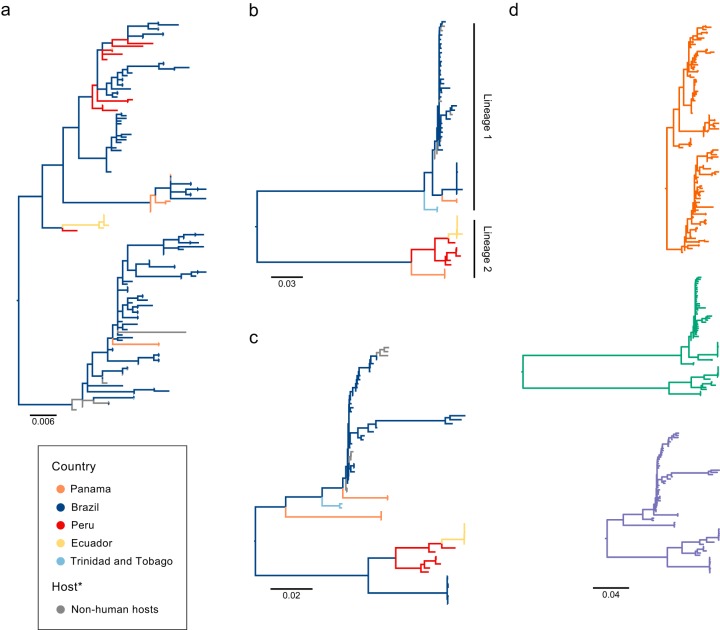
Maximum-likelihood trees for the three OROV genome segments. (a) Small (S) segment phylogeny. (b) Medium (M) segment phylogeny. (c) Large (L) segment phylogeny. Branches are color coded by the country of origin for each sample, with gray samples indicating sequences obtained from nonhuman hosts and vectors. (d) A scaled comparison of the three segment phylogenies highlights the different node depths between all trees.

The estimated ML phylogenies for each OROV genome segment are shown in Fig. 1 and in Fig. S2 in the supplemental material. An interactive phylogenetic representation that includes additional information such as sampling location is available through Microreact (29) (URL links are provided in Table S2). The phylogenies show spatially structured virus populations as evidenced by the clustering of taxa according to country of isolate collection. In all three phylogenies (Fig. 1a to c), our new sequences from Ecuador (yellow) cluster together in a single monophyletic group (with 100% bootstrap support, Fig. S2) that descends from OROV sequences from Peru (red), suggesting the Ecuadorian sequences represent an unreported outbreak in 2016 that is related to cases observed in Peru in the middle to late 2000s. The large (L) and medium (M) segment trees (Fig. 1b and c) contain long internal branches and distinct lineages. Sequences from Panama (orange) are found in two distinct clusters, while Brazilian sequences (blue) are split into two distinct groups. One Brazilian group corresponds to the 2009 OROV outbreak in the municipality of Mazagão (Amapá state) in northern Brazil (7), while the other contains sequences from isolates obtained between 1960 and 2006 from seven different states (Acre, Amapá, Amazonas, Maranhão, Minas Gerais, Pará, and Rondônia) (30). Most of these clusters are supported by high bootstrap scores (>95%), but there is less consistent and sometimes limited support for phylogenetic structure *within* clusters (Fig. S2b and c). The OROV small (S) segment phylogeny (Fig. 1a) contains more sequences but has less phylogenetic structure (i.e., the internal branches are shorter, and several basal nodes have weak bootstrap support). Geographical clustering is also less evident; there are three well-supported clusters of sequences from Brazil or Brazil/Panama, and the Ecuadorian sequences are again found in a strongly supported cluster descended from the Peruvian isolate IQE7894, but the remaining sequences from Peru cluster elsewhere in the tree (Fig. 1 and Fig. S2a).

The trees for the different segments show considerable topological differences, suggesting the likelihood of reassortment events in the viral genome. Pairwise comparisons between segment phylogenies (Fig. S3) indicate that the L segment of the 2009 Mazagão outbreak sequences has arisen through a reassortment event. There are also widespread topological differences between the S segment and the L and M segments (Fig. S3), particularly for the Brazilian sequences (which cluster in robust monophyletic clades in the L and M segment trees but as multiple clusters in the S segment phylogeny). This suggests that OROV might reassort frequently; however, the low node support for the S segment could also represent phylogenetic uncertainty rather than differing evolutionary history. Furthermore, a comparison of the three segment trees drawn on the same scale shows widely differing tree lengths (Fig. 1d).

**FIG 2:**
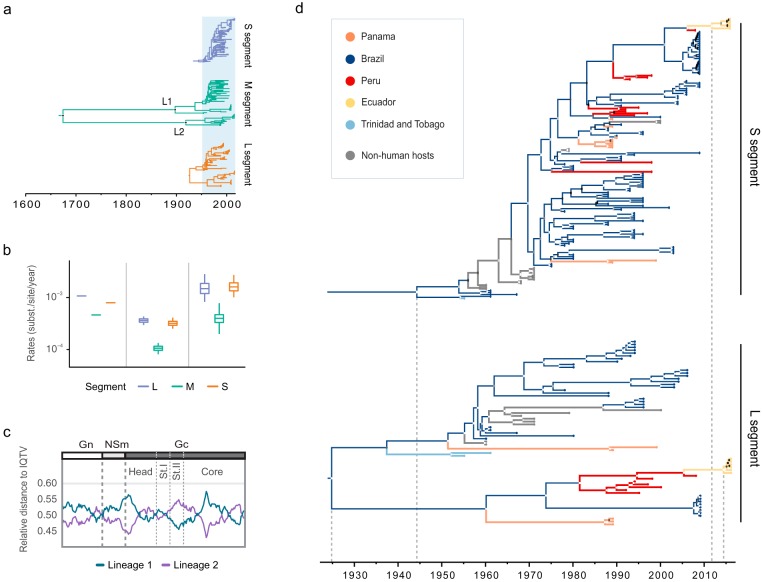
Time-calibrated and evolutionary rate analysis of the OROV genome. (a) A comparison of the three segments reveals an older common ancestor for the M segment, resulting in the divergence of two lineages that appear to diversify in the early to mid-1900s (lineage 1 and lineage 2 are indicated as L1 and L2 in the figure). (b) Estimated evolutionary rates of the segments. (c) Sliding window estimation of the relative genetic distance of each of the two M segment lineages (shown in orange and blue) to Iquitos virus (IQTV), used as an outgroup. Points of the sequences where the closest genotype to the outgroup changes could be interpreted as recombination events with unsampled sequences. (d) An analysis of the younger segments, S and L, reveals emergence date estimates for the virus in the early to middle 20th century, and shows relatively consistent estimates for the TMRCA of the new Ecuadorian sequences in the early 2010s. The posterior probabilities (PP) of each node is shown based on a gray-scale color scheme (0.0 PP = black to 1.0 PP = white).

The evolutionary timescale for OROV in America, estimated using a Bayesian phylogenetic molecular clock approach (relaxed uncorrelated molecular clock [UCLD] model), yielded highly variable dates for the most recent common ancestor (TMRCA) for each segment. The origins of the S and L segments can be traced back to the early to mid-20th century, while the M segment shows long internal branches and an older mean root age, dating back to approximately 1608 (95% highest posterior density [HPD], 1116 to 1924) (Fig. 2a). The estimated TMRCA of the S segment is approximately 1940 (95% HPD, 1916 to 1955), while that of the L segment is slightly older, around 1919 (95% HPD, 1854 to 1955). It is also noteworthy that the M segment splits into two distinct lineages (Fig. 2a), observed both in the ML and the Bayesian phylogenies. Lineage 1 represents an eastern-central clade that includes the earliest OROV case in Trinidad and Tobago, sequences obtained from patients in Panama between the late 80s and late 90s, and the entirety of the Brazilian genetic diversity of the virus. Lineage 1 is estimated to have emerged around 1887 (95% HPD, 1804 to 1945). On the other hand, lineage 2 contains sequences from western South America, including sequences from Panama (from the late 1980s), Peru and Ecuador, with an estimated origin around 1905 (95% HPD, 1841 to 1962). The estimated mean evolutionary rate of the M segment appears to be significantly lower than that of other segments (mean rates = 1.75 × 10^−3^ substitutions/site/year for segment S, 3.65 × 10^−4^ substitutions/site/year for segment M, and 1.64 × 10^−3^ substitutions/site/year for segment L). We also attempted to estimate evolutionary rates estimated separately for each lineage within the M segment phylogeny. A systematic comparison of different molecular clock methods was used to evaluate the reliability of the lineage-specific estimates (Fig. S4). While the estimated rates for Lineage 1 are similar to those for the whole M segment, the rate estimates for lineage 2 are highly uncertain and also inconsistent among methods and models (Fig. S4). Thus, the small sample size of lineage 2 renders the estimate for that lineage unreliable and prevents us from concluding the lineages evolve at different or similar rates. The systematic comparisons in Fig. S4 and 2b also show that the relative rates of the segments are broadly consistent (i.e., L is fastest, S is intermediate, and M is slowest; Fig. 2b and Fig. S4), despite differences in their estimated absolute rates. The UCLD clock remains the most reliable and realistic model choice due to the high among lineage rate variation we observe (coefficient of variation >1 for all segments) and low *R*^2^ values in the linear regression analyses (Fig. S1).

In order to explore whether these differences in evolutionary rates might result from rate heterogeneity between the M segment lineages, we calculated the mean relative genetic distance of Lineage 1 and 2 to a closely related outgroup, Iquitos virus (IQTV), using a sliding window approach. In each window we divided the mean genetic distance of each lineage to the outgroup by the sum of the genetic distances for both lineages, resulting in a relative genetic distance to IQTV (see supplementary R Script at https://github.com/BernardoGG/OROV_America/blob/master/OROV_SimPlots.R). These relative distances are the fraction of the cumulative genetic distance of both lineages to the selected outgroup (and accordingly sum to 1). Different regions of the M segment exhibit different relative distances to the outgroup, ranging between 0.58 and 0.42 (as expected for two lineages that are roughly equidistant to the outgroup). The lines cross where one lineage becomes relatively less divergent to the outgroup, and several such events occur between the NSm and Gn reading frames, within the Gn reading frame, and the core region and head domain of the Gc reading frame (Fig. 2c). In summary, we find no evidence of strong intrasegment evolutionary rate variation between the two lineages.

In the S segment phylogeny, samples obtained through the 1950s and 1960s are located at the base of the tree. The tree splits into two cocirculating clusters around 1969 (95% HPD, 1964 to 1973). Both clusters include sequences mostly from central Brazil and Panama, and one of the clusters suggests multiple introductions to Peru as early as the mid 1970s. The 2009 Mazagão outbreak and the 2016 Ecuador sequences arise from one of these Peruvian clusters, with the latter sharing a common ancestor around late 2011 (95% HPD, 2009 to 2015) (Fig. 2d). On the other hand, the L segment tree shows two divergent lineages that split sometime in the early to mid-20th century (95% HPDs, 1900 to 1953 and 1918 to 1979) and more closely match the M segment lineage structure. One of these L segment lineages includes most of the Brazilian diversity (the Mazagão sequences are distinct and cluster in the other L segment lineage) and a cluster of sequences from Panama. The other includes a second cluster of Panama sequences and all of the Peruvian diversity (Fig. 2d). The Ecuadorian sequences are again descended from Peruvian strains, with a TMRCA in early 2014 (95% HPD, 2011 to 2016). This date is similar in the other segments (the estimated Ecuador TMRCA is mid 2011 in the M segment; 95% HPD, 2008 to 2015). These results suggest a recent introduction of OROV to northern Ecuador with no reassortment events leading to its emergence.

### Selection analyses.

We undertook a screen for sites under selection using different models implemented in the HyPhy framework (31). Evidence for both pervasive and episodic selection was found in different genes using different methods (Table 1). Pervasive selection (selection that occurs persistently along the whole phylogeny) was detected in the S segment. Individual sites under pervasive selection were identified in the M segment alignments: for the Gn protein one site was found to be evolving under positive selection (by the SLAC and FEL methods), for the NSm protein one site was identified (by the FEL method), and for the the Gc protein two sites were found to be under selection (by the FEL and FUBAR methods). Sites evolving under pervasive selection were also identified in the S and L segments (one site in each) by the FUBAR method (Table 1). Episodic adaptive selection (selection that occurs heterogeneously across the tree branches) was detected in all alignments under the mixed effects model (MEME) (32). In total, MEME identified six sites under selection in the RNA-dependent RNA polymerase (RdRp), seven sites under selection in the M segment polyprotein, and one site under selection in the nonstructural N protein. Of these sites, only four were identified by two or more methods: codon 66 of the Gn scaffold protein, codon 86 of the NSm nonstructural protein and codons 269 and 442 of the Gc spike protein (Table 1). We note that three of these four sites feature more than two alleles, and the frequencies of the most common allele range from 0.44 to 0.73 (Table S3). This episodic selection is made evident when comparing the difference between synonymous and nonsynonymous rates per site on the M segment, where, despite a low proportion of sites identified as evolving under selection (Fig. S5a), each lineage shows different sites with higher nonsynonymous substitution rate (Fig. S5b).

**TABLE 1:** Evidence for adaptive selection on the three genome segments of the OROV genome assessed using different models^*a*^

Segment	Gene	Length (nt)	Pervasive selection								Episodic selection			
			Gene-wise (BUSTED)		Site-wise (SLAC)		Site-wise (FEL)		Site-wise (FUBAR)		Site-wise (MEME)		Branch-site model (aBSREL)	
			*P* < 0.05	Mean *dN*/*dS*^*b*^	*P* < 0.05	Sites	*P* < 0.05	Sites	*P* < 0.05	Sites	*P* < 0.05	*n*	*P* < 0.05	Sites
S	N/Ns	693	Yes	0.071	No	–	No	–	Yes	1089	Yes	2	No	–
M	Gn	918	No	0.055	Yes	**66**	Yes	**66**	Yes	**66**, 274	Yes	**66**	No	–
	NSm	525	No	0.114	No	–	Yes	**86**	Yes	**86**	Yes	**86**	No	–
	Gc	2,817	No	0.050	No	–	Yes	**269**, **442**	Yes	**269**, **442**	Yes	71, 106, 145, **269**, **442**, 645, 842, 879	No	–
L	RdRp	6,756	No	0.027	No	–	No	–	Yes	230	Yes	237, 1,089, 1,394, 1,397, 2,048, 2,085	No	–

a Sites identified by more than one method are highlighted in boldface. nt, number of nucleotides; Sites, number of sites; –, not applicable.

b Estimated as weighed ω mean values from the unconstrained model.

### Structural analysis of OROV proteins and sites under selection.

**FIG 3:**
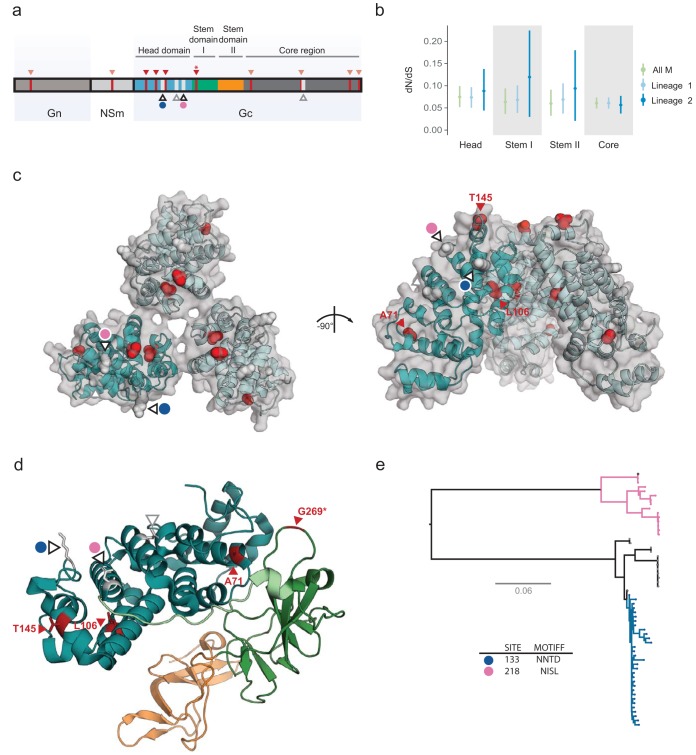
Structure-based mapping of sites identified under selection and previously unreported N-linked glycosylation sites onto the Gc protein. (a) Schematic of the OROV M segment polyprotein, which includes the Gn and Gc components of the viral membrane proteins. The Gc protein is subdivided into a head domain (blue), two stalk domains (green and orange), and a C-terminal region, which encodes a putative fusion loop (dark gray). Sites identified as being under positive selection using the MEME approach are shown in red (lighter colored arrows indicate sites falling outside the Gc head and stem domains). Site 269 was identified as being under positive selection through multiple approaches and is marked with an asterisk. All N-linked glycosylation sites are shown in white, and the two previously undescribed sites are highlighted in blue and pink. (b) Estimated *dN*/*dS* ratio of each domain in Gc, obtained for the whole M segment, and for each M segment lineage. (c) Crystallographically observed trimeric spike structure of the Gc head domain, with sites under positive selection shown in red. The cartoon representation of each chain is embedded in a transparent surface representation (gray). Glycosylation sites are shown as in panel a. (d) Sites under positive selection and residues corresponding to N-linked glycosylation sites mapped to a composite model generated from previously reported OROV Gc (head domain, PDB ID 6H3X) and SBV Gc (stem domains, PDB ID 6H3S) crystal structures. The OROV head domain is shown in blue, with the C terminus loop residues shown in light green (taken from the SBV head domain) and SBV stem domains I and II shown in green and orange, respectively. (e) Putative N-linked glycosylation sites identified in this study are mapped onto the phylogeny of the M segment, showing the independent presence the two sites in the two M segment lineages.

Viral glycoproteins are important targets of positive selection and drivers of virus adaptation due to their direct interactions with the host immune system (33). In orthobunyaviruses, glycoproteins are expressed as a polyprotein, encoded by a single open reading frame (ORF) of the M segment that is expressed and posttranslationally cleaved into the Gn, NSm (nonstructural), and Gc proteins, respectively. The main component of the OROV particle spikes is Gc, which is composed of four main domains: a head domain, two stem domains, and a core region (20). We identified eight sites under selection in Gc overall (four of which fall within the head and stem domains) and two putative, previously unreported N-linked glycosylation sites (Fig. 3a). Estimated *dN*/*dS* ratios are similar for each domain (Fig. 3b); hence, we find no evidence for significantly different selective pressures among domains. However, if *dN*/*dS* ratios are estimated separately for the two M segment lineages, then we obtain higher *dN*/*dS* estimates for the head and stem domains of lineage 2, although the large confidence limits mean these differences are not significant (Fig. 3b).

We mapped all the sites identified as being under positive selection to the available atomic structures of the OROV Gc head domain and the closely related SBV stem domains (Fig. 3b and c). This analysis reveals that three sites under selection (corresponding to residues A71, L106, and T145) are located at alpha-helixes in the head domain, and one (residue G269) occurs at a loop connecting the stem domain I N terminus to the head domain (Fig. 3c). Although A71 is buried and residue L106 is only partially accessible from within the protein structure, residues T145 and G269 are completely accessible (Fig. S6), with the former located at the top of the trimeric spike structure (Fig. 3b). Structure-based mapping of the residues equivalent to T145 in BUNV and LACV suggests that this residue could help to facilitate the formation of a closed trimeric interface (20), so substitutions at this location may affect the protein-protein interface and change the antigenic surface of the spike complex.

In addition to the sites under positive selection, we identified two previously unreported N-linked glycosylation sequons at residues 133 (motif NNTD) and 218 (motif NISL). Both of these putative glycosylation sites are located on the Gc head domain (Fig. 3a), and they do not co-occur in any of the sequences, which supports the hypothesis that they may be alternative motifs serving similar functional roles. Furthermore, these sites appear to be phylogenetically associated with the different M segment lineages, with motif NNTD being exclusive to lineage 1 and motif NISL occurring exclusively in lineage 2 (Fig. 3d). Based on shared ancestry, it is likely that the motif NNTD appeared in a Brazilian common ancestor that excludes the Mazagão outbreak sequences, since this motif is not found in either the Panama or Trinidad sequences belonging to lineage 1.

Both the putative fusion loop in the C-terminal region of the Gc protein (34) and two previously reported N-glycosylation sites were conserved among all analyzed OROV sequences (3).

## DISCUSSION

The evolutionary genomics of OROV in the Americas appears to be driven by a complex combination of factors that are associated with viral life cycle and the nature of the OROV genome. Phylogenetic analysis of the three OROV genome segments reveals different evolutionary histories, with a stronger topological concordance between the M and L segments, than between those two segments and the S segment. Many of these incongruencies likely represent the different evolutionary histories of each segment, arising from reassortment. The occurrence of reassortment events appears to be relatively common for OROV in the Americas and may be currently underestimated because (i) there are likely to be many undiscovered OROV lineages and (ii) the virus likely circulates widely in one or more as-yet-uncharacterized reservoir populations in the Amazonian forest. Although we found similarities between the M and L segment phylogenies, a previous study of the Bunyamwera group has suggested linkage between the L and S segments (35), and different reassortment patterns have been reported for other bunyaviruses such as La Crosse virus and snowshoe hare virus (36). The reassortment of OROV is in line with the observation of naturally occurring reassortant viruses (23, 24), a phenomenon that has been linked to the coinfection of hosts or arthropod vectors. The latter provide a good opportunity for reassortment to occur, since an infected vector can feed on a second vertebrate host infected with a distinct viral strain within a 2- to 3-day time window and become coinfected (2). On a broader scale, reassortment events could play a crucial role in the evolution of bunyaviruses in general, and it has been suggested that an increasing number of the recently described species are reassortants (37).

Molecular clock phylogenetic analyses can be used to infer the emergence times of pathogens and the timescales of outbreaks (38). Some of these tools are particularly applicable to heterochronous data sets, in which sequences from rapidly evolving populations have been sampled longitudinally through time. For molecular clock analyses to be reliable, samples collected at different time points must accumulate sufficient genetic differences to be distinct from one another (39) (i.e., contain sufficient “temporal signal”). A combination of factors may affect the accuracy of estimated TMRCAs. Our analyses of the S and L segments suggest that OROV first emerged in the early to mid 1900s, a more recent estimate than previously reported (late 1700s, inferred from S segment sequences) (26). This difference may reflect the fact that our analysis incorporates more sequences, or it may reflect differences in the evolutionary models or prior distributions used in Bayesian phylogenetic inference. It should be also noted that the aforementioned earlier study (26) did not report a test for temporal signal in the data set; the absence of this signal could affect the precision of estimates.

A key factor of OROV evolutionary dynamics is the distinction between two well-supported lineages for the M segment. We estimate that these two lineages split more than two centuries before the estimated TMRCAs of the other segments. The split between lineages 1 and 2 is also reflected in the L segment phylogeny, where two well-supported lineages that mostly coincide with the M phylogeny are observed, with the exception of the 2009 Mazagão outbreak reassortant group (see Fig. 2; see also Fig. S2 and S3 in the supplemental material).

**FIG 4:**
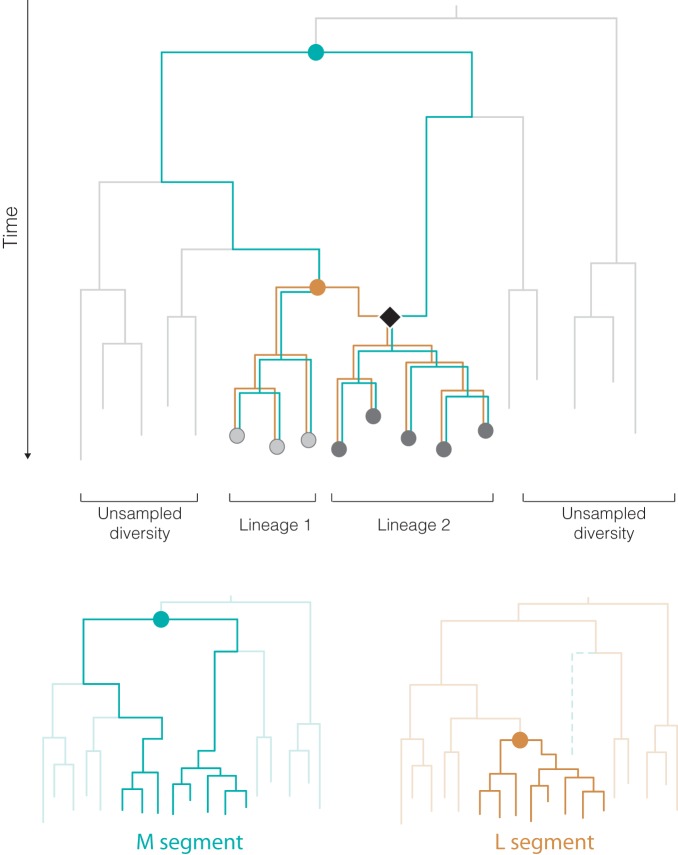
Model of OROV genome evolution and reassortment that can resolve the inconsistent TMRCA estimates of the L and M segments. The upper panel depicts a simplified ancestral graph that describes the joint ancestry of the L (orange) and M (teal) segments. Sampled sequences are represented by dark and light gray circles at the tree tips. These are grouped into lineages 1 and 2, which correspond to the two main lineages in the M and L phylogenies (see Fig. 2). The light-gray branches represent hypothesized, highly diverse, unsampled OROV lineages. The graph contains one reassortment event (black diamond), resulting in the ancestor of lineage 2 in the M segment being descended from a divergent, unsampled lineage (alternatively, the reassortment might have occurred on the branch ancestral to lineage 1). As a result, the TMRCA of the L segment phylogeny (lower right panel; orange circle) exists substantially earlier of the M segment (lower left panel; teal circle). For simplicity, other more recent reassortment events between lineages 1 and 2 are not shown (i.e., the 2009 Mazagão outbreak clade).

We sought to explain the substantially older TMRCA of the M segment compared to the other two segments, despite the fact that all segments share overlapping sets of strains. The temporal signal we observed (Fig. S1) suggests that the roots of the M and L segment phylogenies represent different ancestral viruses and that the discrepant TMRCAs are not the result of estimation error. How can this conclusion be reconciled with the topological similarities between the M and L segment phylogenies? We suggest that these results could be explained by hypothesizing that the ancestor of one of the M segment lineages underwent a past reassortment event with an unsampled, divergent OROV lineage (Fig. 4). At present, it is not possible to determine which of the two lineages actually underwent reassortment (for visualization in Fig. 4, lineage 2 was chosen randomly). Under this hypothesis, the TMRCAs of segments M and L can differ substantially, and yet their phylogenies remain largely congruent. This hypothesis posits that a large diversity of OROV in South America exists, which is currently unsampled and among which reassortment is common.

The lower evolutionary rate of the M segment is also unexpected, given the tendency of bunyaviral surface proteins to evolve faster than the nucleocapsid and nonstructural proteins (40, 41). These differences are hard to reconcile with the available data, but the reliability of the temporal signal should be considered. A systematic comparison of different molecular clock approaches indicates a lineage 2-specific rate cannot be reliably estimated. We conclude that most of the temporal signal in the M segment comes from lineage 1 sequences, due to the small sample size and high uncertainty of lineage 2 rate estimates. More OROV genome sequences (especially for lineage 2) are clearly needed to provide a robust understanding of whether the molecular evolution of the two lineages truly differ.

The adaptation of an emerging virus to new hosts can affect the likelihood of future outbreaks and is a useful phenomenon to explore. While computational evolutionary analysis can usefully indicate the presence of positive selection and molecular adaptation in virus genomes, sequence data alone cannot resolve the functional and biological context of selected sites. Further, different methods for detecting selection do not always give the same results; hence, conclusions benefit from considering the consensus of results generated by multiple models and methods (42). Three different models all indicated the positive selection acting on codon 66 of Gn (Table 1). The N terminus of closely related Bunyamwera virus (BUNV) Gn protein is predicted to localize outside the viral particle (43) and function as a scaffold for the trimeric Gc spike (19). The residue encoded by codon 66 falls within this predicted extramembrane domain, potentially exposing it to the host immune system. Unlike other bunyaviruses, such as phleboviruses (44, 45) and hantaviruses (46, 47), it is unlikely that the Gn is large enough to extend from the virion membrane and shield the fusion loops of the cognate Gc. Thus, while amino acid changes are unlikely to affect fusion loop shielding, it is possible that selective forces acting on Gn could just alter the stability of the Gn-Gc lattice during cell entry.

We also detected positive selection on codon 86 of the NSm. The function of this protein among the orthobunyaviruses is variable, ranging from being essential for viral assembly in BUNV (particularly the N-terminal hydrophobic domains) (48) to being nonessential for viral growth in OROV (7) and other related pathogens such as Maguari virus (49). In Rift Valley fever virus (RVFV), NSm is nonessential for viral replication (50, 51) but plays a role in the suppression of apoptosis in infected cells (52, 53). Other potential roles for NSm in different bunyaviruses have been proposed (54), including a role in the infection of arthropod vectors (55). The reason for the adaptive evolution in OROV NSm detected here is therefore unclear.

The N-terminal head and stem domains of the orthobunyaviral Gc spike are the only regions for which atomic structures are available (20), making structure-based mapping of sites feasible. Although little is known about the host immune response to OROV, SBV Gc has been shown to be a target of antibody-mediated immune responses following infection (56) and is therefore likely to be under host selective pressure. Codons 269 and 442 (identified as evolving under positive selection) encode residues located in different domains of the Gc protein, in the first stem domain and core region, respectively. We mapped codon 269 to a loop at the N terminus of the stem domain I, which connects to the head domain. Although we cannot discount a potential role in shielding the fusion loop in the core region, crystallographic analysis and comparison of this region with other reported orthobunyavirus Gc structures suggests that this loop may act as a flexible hinge that permits fusogenic rearrangements of the spike complex (20). Furthermore, and in line with the hypothesis that this region is important in stabilizing the mature spike complex, an identified neutralizing antibody against SBV directly interacts with residues closer to the C terminus of the protein’s head domain, potentially disrupting Gc-mediated trimerization of the spike protein, as presented on viral particles (20). A similar immunogenic mechanism could play a role in the evolution of residue G269, rendering it a potential target of the neutralizing antibody response. Of the residues that were identified as evolving under diversifying selection by the MEME approach alone, codon 145 encodes a residue that is solvent accessible and mapped to an alpha-helix in the head domain (20), specifically the tip of the trimeric spike complex. The latter site could also constitute an antibody epitope, even if the evidence for this is presently weak (only one method identifies this residue as evolving under positive selection). Furthermore, if this region includes an epitope, the acquisition of N-linked glycosylation may mask the protein surface, similar to the “glycan shields” described for Old World arenaviruses and HIV-1 (57, 58). N-linked glycosylation that is not essential for replication but increases virus infectivity of new cells has been described in BUNV (59) and RVFV (60), suggesting that different bunyaviruses could gain and lose glycosylation sites as they evolve, as a mechanism to mask spike epitopes from the humoral immune system. Mechanisms other than immune selection might play a role. For example, mutations in the E1 protein of Chikungunya virus affect its infectivity in different vector species (A226V increases infectivity in Aedes albopictus compared to Aedes aegypti) (61). The geographical spread of our isolates means that OROV variation could be associated with different host or vector species in South America; however, current data are insufficient to explore this idea further.

Our report incorporates new OROV genomes obtained from febrile patients in the province of Esmeraldas in northern Ecuador, representing the first direct detection and whole-genome sequencing of the virus in the country (14). Previous prospective studies have suggested that OROV may circulate in both the coastal and Amazonian provinces of Ecuador (18, 62) but failed to directly detect or isolate the virus. Our sequences probably represent an undetected outbreak of OROV on Ecuador’s northern coast, which may also include the southern coast of Colombia. Our phylogenetic analyses show that the Ecuadorian isolates form a single lineage that is closely related to viruses circulating in Peru, indicating a contiguous expansion of the known area of OROV circulation. The Ecuadorian isolates carry a distinct M segment that is shared with sequences from Peru and Panama, suggesting the presence of a divergent OROV lineage in the western, Pacific-bordering countries of South America.

OROV continues to be a neglected tropical disease and the numbers of genetic sequences for the virus are still limited. The genetic data that are available tend to be biased toward the shorter S segment, which has lower phylogenetic resolution and provides only a partial picture of OROV evolution. Available sequences are for the most part limited to viral diversity detected during human outbreaks and therefore fail to capture the dynamics of OROV in reservoir host species and vector populations. The generation of whole OROV genomes, for example, those generated locally using new rapid and portable sequencing technologies (63, 64), and the broadening of sampling to other countries and host species are necessary to improve our understanding of the extent and nature of OROV transmission and the importance of reassortment for the evolution of this pathogen.

## MATERIALS AND METHODS

### Sampling and sequencing of OROV isolates from Ecuador.

Six strains of OROV were isolated independently via one passage in Vero cells from febrile patient sera sampled in 2016 in the Esmeraldas province of Ecuador (27). RNA was extracted, and six complete genome sequences were generated using a metagenomic approach as described previously (14). Briefly, cDNA was prepared using a sequence independent single primer amplification approach (65), cDNA sequencing libraries were prepared using the Nextera XT kit (Illumina), and sequencing was performed on a MiSeq (Illumina) using 150-nucleotide paired-end runs. Reads were mapped to reference sequences using BWA MEM (66). The final consensus sequences were generated using Quasibam (67). We visually inspected each data set for the occurrence of single nucleotide variants within each patient; limited variation per site was found for all isolates (detailed in File S1 in the supplemental material). The study was approved by the bioethics committee of Universidad San Francisco de Quito, and all patients provided written consent indicating that they agreed for their samples to be tested for additional pathogens.

### Data sets.

In addition to the Ecuadorian sequences, we collected all available sequences for the three OROV genome segments from GenBank at the National Center for Biotechnology Information. We then excluded all sequences that were shorter than a threshold segment length (specifically, <6,500 nucleotides long for L, <4,200 nucleotides long for M, and <693 nucleotides long for S). These GenBank sequences represent the genetic diversity of OROV from outbreaks in Trinidad and Tobago, Panama, Peru, and Brazil and cover the complete sampled history of the virus from the mid 1950s to the late 2000s, with the latest outbreak in Brazil reported in 2009 (7). The data sets were compiled into individual alignments for each of the three viral genome segments (denoted L for large, M for medium, and S for small). A complete list of the GenBank accession numbers of all sequences used is presented in Table S1.

Additional data for the OROV sequences included the sampling date (with varying precision of year, month, or exact day, depending on the sequence), the identity of the host species (most samples were obtained from human patients, while a few were obtained from potential arthropod vectors and mammalian reservoir species), and the location of each sample (state or department of origin was used for the samples from Brazil and Peru, while only the country of origin was available for the Panama sequences; the samples from Ecuador and Trinidad and Tobago were obtained from a single location). No exact sampling date is available for the Ecuadorian sequences, but sampling was conducted between the months of April and May of 2016 (Sully Márquez, unpublished data).

Before sequence alignment, the untranscribed terminal repeats of each segment were removed, and the ORF of each segment was identified (only the largest ORF of the S segment, corresponding to the N gene, was analyzed). Sequences were then aligned using the MUSCLE algorithm, as implemented in Geneious 9.0.5 (68), and the alignments were manually checked and edited to ensure they were codon aligned. The L alignment included 59 published sequences plus the six new sequences from Ecuador. The M segment alignment included 58 published sequences and the six Ecuadorian sequences. Finally, the S segment alignment included 143 published sequences plus the six Ecuadorian sequences.

### Molecular phylogenetics and molecular clock analyses.

All three alignments were scanned for recombinant sequences using the suite of methods implemented in RDP4 (RDP, BOOTSCAN, MAXCHI, CHIMAERA, 3SEQ, GENECONV, LARD, and SISCAN) (69). Sequences identified as recombinant (i.e., evidence for recombination was found by at least four methods) were removed from all further analyses (only two M segment sequences were excluded). For each alignment, a molecular phylogeny was estimated using the maximum-likelihood (ML) approach implemented in RAxML 8.0 (70). ML trees were estimated using a general-time-reversible substitution model with a gamma-distribution model of among-site heterogeneity. Phylogenetic node support was assessed using a nonparametric bootstrap approach with 100 replicates. No outgroup sequences were used, and all trees were midpoint rooted. A visual comparison of the tree topologies (after collapsing some nodes to facilitate visualization) and bootstrap scores for each segment was used to evaluate possible OROV reassortment events during its evolutionary history. Well-supported topological incongruencies between trees were interpreted as a potential reassortment event between the corresponding genome segments.

To explore the rates of OROV molecular evolution and to evaluate the temporal signal in each OROV alignment, we correlated tip-to-root genetic distances in the ML tree against the sampling dates of the corresponding sequences, using the approach implemented in TempEst 1.5.1 (71). The regression plots were inspected visually to identify notable outliers, and the linear correlation coefficient of the regression was used as a measure of the degree to which the sequences evolve in a clock-like manner.

We estimated the evolutionary history of OROV outbreaks in South America by constructing time-calibrated maximum clade credibility (MCC) trees for each segment. Each MCC tree summarizes a posterior distribution of phylogenies that were sampled using the Bayesian Markov Chain Monte Carlo approach implemented in BEAST 1.10.1 (72). Trees were sampled under a HKY substitution model with codon-position partitioning and a gamma-distribution model of among-site heterogeneity (this model was chosen following previously reported results [73]), a Bayesian Skyline tree prior (74), and a relaxed uncorrelated molecular clock (UCLD) (75) model. For all molecular clock analyses from this point onward, the outlier sequences identified from the tip-to-root regression plots (Fig. S1) were removed from their corresponding data sets.

Our ML and TempEst analyses (above) revealed two monophyletic lineages in the M segment phylogeny, one with a weak temporal signal (correlation coefficient = 0.66) and one with a stronger signal (correlation coefficient = 0.94; see Results and Fig. S1). These lineages were separated by long internal branches that might have unexpected effects on the estimation of evolutionary rates and the age of the root of the tree (76). Therefore, the molecular clock analysis of the M segment was performed as a two-step process. First, we estimated a time-calibrated tree separately for the two M lineages and noted the estimated time of the most recent common ancestor (TMRCA) of the larger lineage 1 (the very small sample size of lineage 2 means that it is unlikely to generate a robust evolutionary timescale; see results below and Fig. S4). We then performed a second analysis of the whole group M phylogeny, in which this TMCRA estimate was used as an additional calibration point (i.e., as an informative prior on the date of the common ancestor of lineage 1). Similarly, mean evolutionary rates were coestimated with phylogeny for each of the three segments using the UCLD clock model and subsequently compared. We further repeated this analysis separately for M segment lineages 1 and 2. Additional analyses using a less-realistic strict molecular clock were also performed for model comparison purposes. All XML files are available via GitHub (https://github.com/BernardoGG/OROV_America).

### Selection analyses.

The ORF alignments of the S and L segments, as well as those of the individual proteins of the M segment ORF (Gn, NSm and Gc), were tested for evidence of natural selection using the various methods implemented in HyPhy (31), implemented by the Datamonkey 2.0 server (77). Statistical tests were used to evaluate evidence for both pervasive and episodic selection in the OROV genome and to evaluate selection across whole genes, as well as at specific codons. Analyses of pervasive selection were performed using three methods: (i) the Branch-site Unrestricted Statistical Test for Episodic Diversification (BUSTED) method, which tests for gene-wise selection and estimates a mean *dN*/*dS* ratio for each gene (78), (ii) the Single-Likelihood Ancestor Counting (SLAC) method, and (iii) the Fixed Effects Likelihood (FEL) method. The latter two approaches were used to infer specific codons under adaptive selection (79). Episodic selection was evaluated using two methods: (i) the Mixed Effects Model of Evolution (MEME), which identifies individual codons under positive selection (32), and (ii) a branch-site model implemented in aBSREL (80), which was used to search for phylogenetic branches under selection. A final analysis was performed using the Fast Unconstrained Bayesian Approximation (FUBAR) (81) method to evaluate the difference between nonsynonymous (β) and synonymous rates (α) per site for each viral segment. All codon positions were numbered relative to the OROV reference strain BeAn19991.

### Structural analysis of OROV proteins and sites under selection.

The availability of high-resolution protein structures for the OROV proteins is limited. We therefore focused efforts on the OROV glycoprotein Gc because (i) N-terminal regions for a number of orthobunyaviral Gc glycoproteins (including the head domain of OROV Gc, PDB ID 6H3X) have been structurally elucidated (20) and (ii) this protein shows an increased number of sites under positive selection compared to other OROV proteins (see Results). The second component of the OROV glycoprotein, Gn, was not analyzed here because no adequate atomic resolution structure is currently available. For Gc, N-linked glycosylation sites were predicted for each virus sequence using the NetNGlyc 1.0 server (www.cbs.dtu.dk/services/NetNGlyc/), which identifies NXT/S amino acid motifs (where X is any amino acid except proline). The sequence evolution of these motifs was subsequently mapped onto the M segment phylogeny using a maximum parsimony approach. To enable structure-based mapping of sites under selection, we created a composite molecular model that encompasses the structurally elucidated N-terminal head of OROV Gc (PDB ID 6H3X) and the stem domains from the closely related Schmallenberg virus (SBV) (PDB ID 6H3S) (20) through sequence-independent structural alignments in PyMOL (82). Since there are no known resolved structures of the orthobunyaviral C-terminal domains (henceforth called the core region), this region was omitted from the structural analysis. Residues in the N-terminal head of the OROV Gc structure that contribute to the crystal packing-induced homotrimeric surface (20) were identified with the PDBePISA server (83). Sites under selection were mapped onto the composite and the trimeric models, and their residue-specific solvent accessibility was analyzed using the ESPript 3.0 server (84).

We explored how the evolutionary history of OROV has been shaped by molecular adaptation of different domains of the Gc protein by estimating the *dN*/*dS* ratio (i.e., the ratio of nonsynonymous to synonymous substitution rates) for each protein domain using the renaissance counting approach (85) implemented in BEAST 1.10.1 (72). These estimates were independently performed for the full set of M sequences and for the two previously described lineages, using an empirical posterior sample of phylogenies comprising >100,000 trees. The 95% HPD credible intervals for the normalized *dN* and *dS* values were summarized using the *coda* package in R (https://cran.r-project.org/web/packages/coda/index.html). The normalized values are the observed count of each substitution type, divided by the expected count of each substitution type under the substitution and evolutionary models and independent of the data.

## Supplementary Material

Supplemental file 1
