# Supplementary material for: Evolutionary Dynamics of Oropouche Virus in South America
Source: J Virol. 2020 Feb 14;94(5):e01127-19. doi: 10.1128/JVI.01127-19 (PMC7022353; doi:10.1128/JVI.01127-19)
Supplement: Supplemental file 1 [file JVI.01127-19-s0001.pdf]

The evolutionary dynamics of Oropouche Virus (OROV) in South America

Bernardo Gutierrez, Emma Wise, Steven Pullan, Christopher Logue, Thomas A. Bowden,

Marina Escalera-Zamudio, Gabriel Trueba, Marcio Nunes, Nuno R. Faria, Oliver G. Pybus

Supplementary Figures

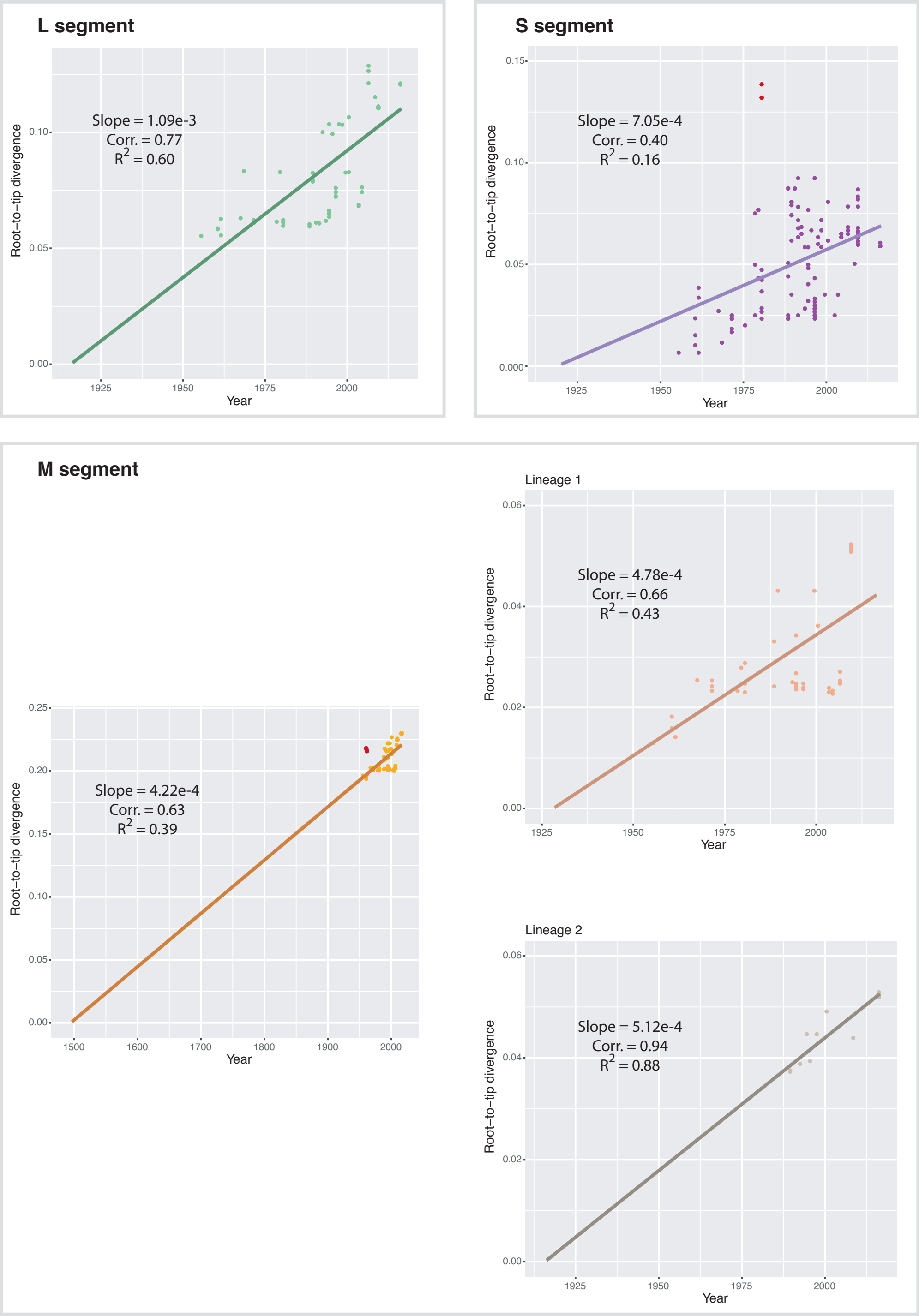

Figure S1 (above). Correlation plots between tip-to-root distances versus sampling estimated in TempEst for the three OROV genome segments. Outliers (determined through visual inspection) are highlighted in red. Sequences corresponding to the points highlighted in red (sequences PA01 and PA03 for the M segment; sequences AM01, AM03 and BeH389865 for the S segment) were excluded as outliers prior to the estimation of the individual plots for each M lineage and all further molecular clock analyses. Regression slope (interpreted as rough estimates of the mean evolutionary rate), correlation coefficients and R^2 values are shown for each plot.

a

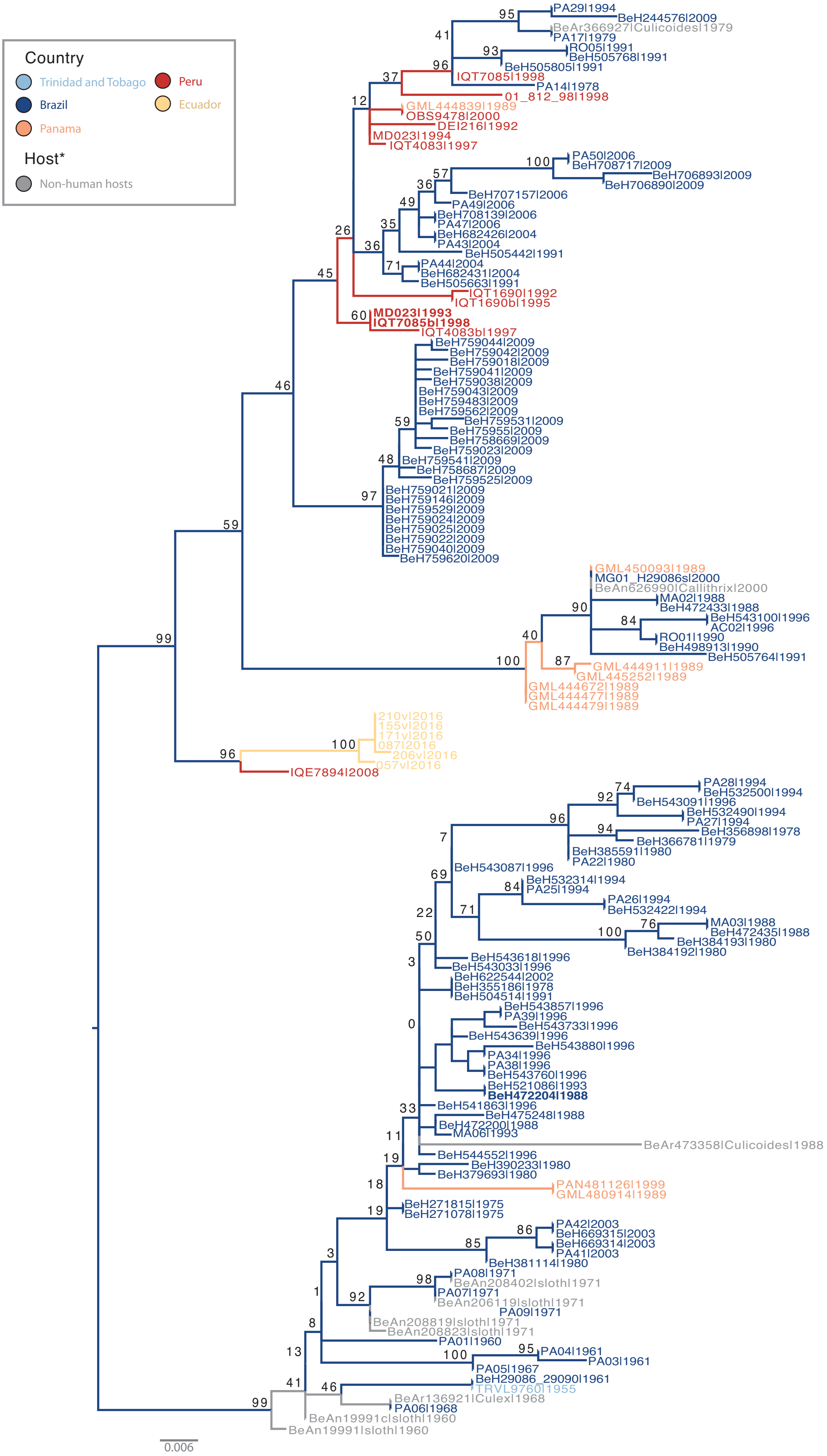

Country
Trinidad and Tobago
Brazil
Panama
Peru
Ecuador
Host*
Non-human hosts
PA29|1994
BeH244576|2009
BeAr366927|Culicoides|1979
PA17|1979
RO05|1991
BeH505768|1991
BeH505805|1991
IQT7085|1998
PA14|1978
01_812_98|1998
GML444839|1989
OBS9478|2000
DE|216|1992
MD023|1994
IQT4083|1997
PA50|2006
BeH708717|2009
BeH706893|2009
BeH706890|2009
BeH707157|2006
PA49|2006
BeH708139|2006
PA47|2006
BeH682426|2004
PA43|2004
BeH505442|1991
PA44|2004
BeH682431|2004
BeH505663|1991
IQT1690|1992
IQT1690b|1995
MD023|1993
IQT7085b|1998
IQT4083b|1997
BeH759044|2009
BeH759042|2009
BeH759018|2009
BeH759041|2009
BeH759038|2009
BeH759043|2009
BeH759483|2009
BeH759562|2009
BeH759531|2009
BeH759555|2009
BeH758669|2009
BeH759023|2009
BeH759541|2009
BeH758687|2009
BeH759525|2009
BeH759021|2009
BeH759146|2009
BeH759529|2009
BeH759024|2009
BeH759025|2009
BeH759022|2009
BeH759040|2009
BeH759620|2009
GML450093|1989
MG01_H29086s|2000
BeAn626990|Callithrix|2000
MA02|1988
BeH472433|1988
BeH543100|1996
AC02|1996
RO01|1990
BeH498913|1990
BeH505764|1991
GML444911|1989
GML445252|1989
GML444672|1989
GML444477|1989
GML444479|1989
210v|2016
155v|2016
171v|2016
087|2016
206v|2016
057v|2016
IQE7894|2008
PA28|1994
BeH532500|1994
BeH543091|1996
BeH532490|1994
PA27|1994
BeH356898|1978
BeH366781|1979
BeH385591|1980
PA22|1980
BeH543087|1996
BeH532314|1994
PA25|1994
PA26|1994
BeH532422|1994
MA03|1988
BeH472435|1988
BeH384193|1980
BeH384192|1980
BeH543618|1996
BeH543033|1996
BeH622544|2002
BeH355186|1978
BeH504514|1991
BeH543857|1996
PA39|1996
BeH543733|1996
BeH543639|1996
BeH543880|1996
PA34|1996
PA38|1996
BeH543760|1996
BeH521086|1993
BeH472204|1988
BeH541863|1996
BeH475248|1988
BeH472200|1988
MA06|1993
BeAr473358|Culicoides|1988
BeH544552|1996
BeH390233|1980
BeH379693|1980
PAN481126|1999
GML480914|1989
BeH271815|1975
BeH271078|1975
PA42|2003
BeH669315|2003
BeH669314|2003
PA41|2003
BeH381114|1980
PA08|1971
BeAn208402|sloth|1971
PA07|1971
BeAn206119|sloth|1971
PA09|1971
BeAn208819|sloth|1971
BeAn208823|sloth|1971
PA01|1960
PA04|1961
PA03|1961
PA05|1967
BeH29086_29090|1961
TRVL9760|1955
BeAr136921|Culex|1968
PA06|1968
BeAn19991c|sloth|1960
BeAn19991|sloth|1960
0.006

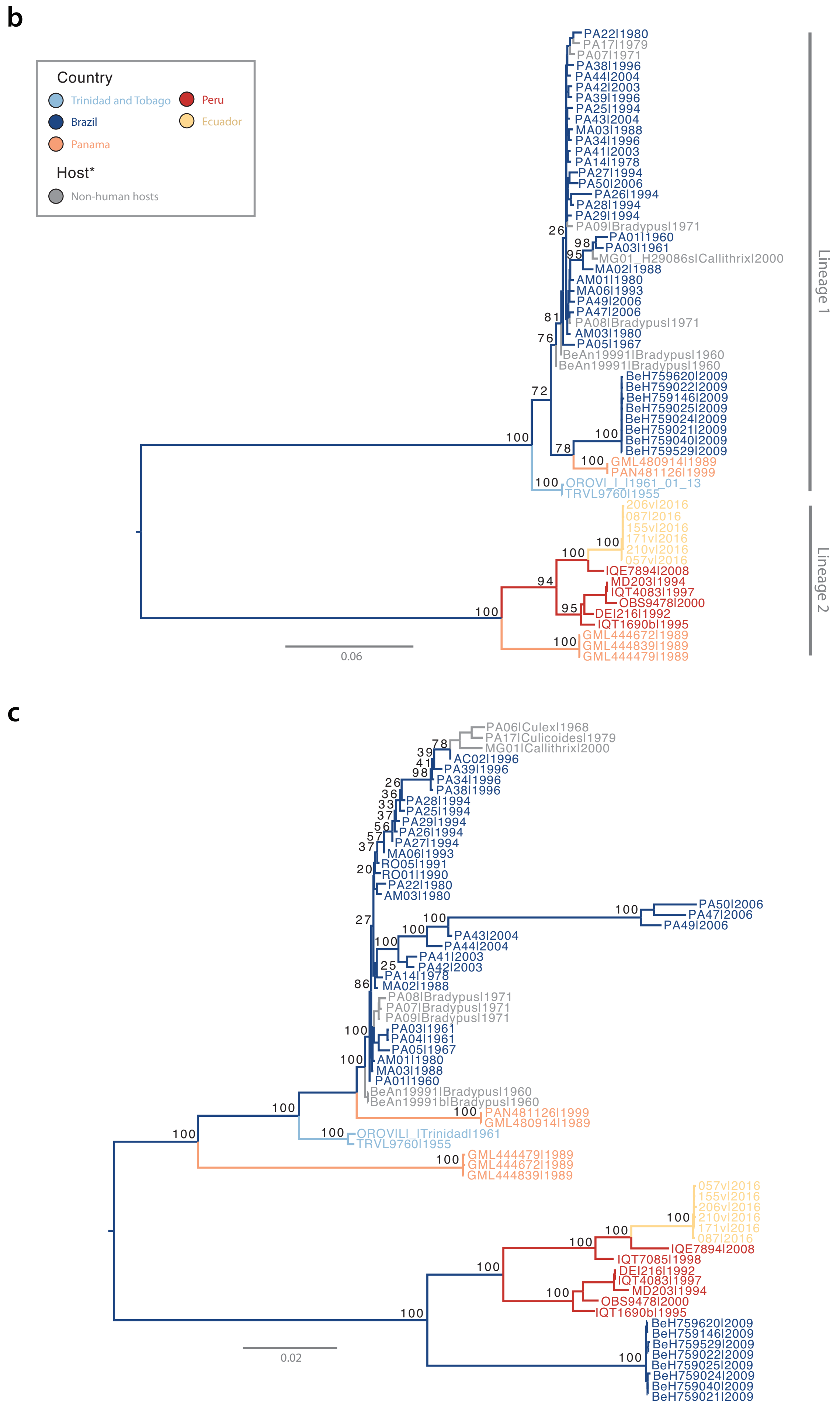
b
Country
Trinidad and Tobago
Brazil
Panama
Peru
Ecuador
Host*
Non-human hosts
PA22|1980
PA17|1979
PA07|1971
PA38|1996
PA44|2004
PA42|2003
PA39|1996
PA25|1994
PA43|2004
MA03|1988
PA34|1996
PA41|2003
PA14|1978
PA27|1994
PA50|2006
PA26|1994
PA28|1994
PA29|1994
PA09|Bradypus|1971
PA01|1960
PA03|1961
MG01_H29086s|Callithrix|2000
MA02|1988
AM01|1980
MA06|1993
PA49|2006
PA47|2006
PA08|Bradypus|1971
AM03|1980
PA05|1967
BeAn19991|Bradypus|1960
BeAn19991|Bradypus|1960
BeH759620|2009
BeH759022|2009
BeH759146|2009
BeH759025|2009
BeH759024|2009
BeH759021|2009
BeH759040|2009
BeH759529|2009
GML480914|1989
PAN481126|1999
OROVI_I_|1961_01_13
TRVL9760|1955
206v|2016
087|2016
155v|2016
171v|2016
210v|2016
057v|2016
IQE7894|2008
MD203|1994
IQT4083|1997
OBS9478|2000
DEI216|1992
IQT1690b|1995
GML444672|1989
GML444839|1989
GML444479|1989
Lineage 1
Lineage 2
0.06
c
PA06|Culex|1968
PA17|Culicoides|1979
MG01|Callithrix|2000
AC02|1996
PA39|1996
PA34|1996
PA38|1996
PA28|1994
PA25|1994
PA29|1994
PA26|1994
PA27|1994
MA06|1993
RO05|1991
RO01|1990
PA22|1980
AM03|1980
PA50|2006
PA47|2006
PA49|2006
PA43|2004
PA44|2004
PA41|2003
PA42|2003
PA14|1978
MA02|1988
PA08|Bradypus|1971
PA07|Bradypus|1971
PA09|Bradypus|1971
PA03|1961
PA04|1961
PA05|1967
AM01|1980
MA03|1988
PA01|1960
BeAn19991|Bradypus|1960
BeAn19991b|Bradypus|1960
PAN481126|1999
GML480914|1989
OROVI_I_|Trinidad|1961
TRVL9760|1955
GML444479|1989
GML444672|1989
GML444839|1989
057v|2016
155v|2016
206v|2016
210v|2016
171v|2016
087|2016
IQE7894|2008
IQT7085|1998
DEI216|1992
IQT4083|1997
MD203|1994
OBS9478|2000
IQT1690b|1995
BeH759620|2009
BeH759146|2009
BeH759529|2009
BeH759022|2009
BeH759025|2009
BeH759024|2009
BeH759040|2009
BeH759021|2009
0.02

Figure S2 (above). Maximum Likelihood trees for the three OROV genome segments. (**a**) Small (S) segment phylogeny. (**b**) Medium (M) segment phylogeny. (**c**) Large (L) segment phylogeny. Tip labels and corresponding branches are colour coded by the country of origin for each sample, with grey samples indicating sequences obtained from non-human hosts and vectors (species indicated in the tip label). Node support from 100 bootstrap replicates is shown for the basal internal nodes of each tree.

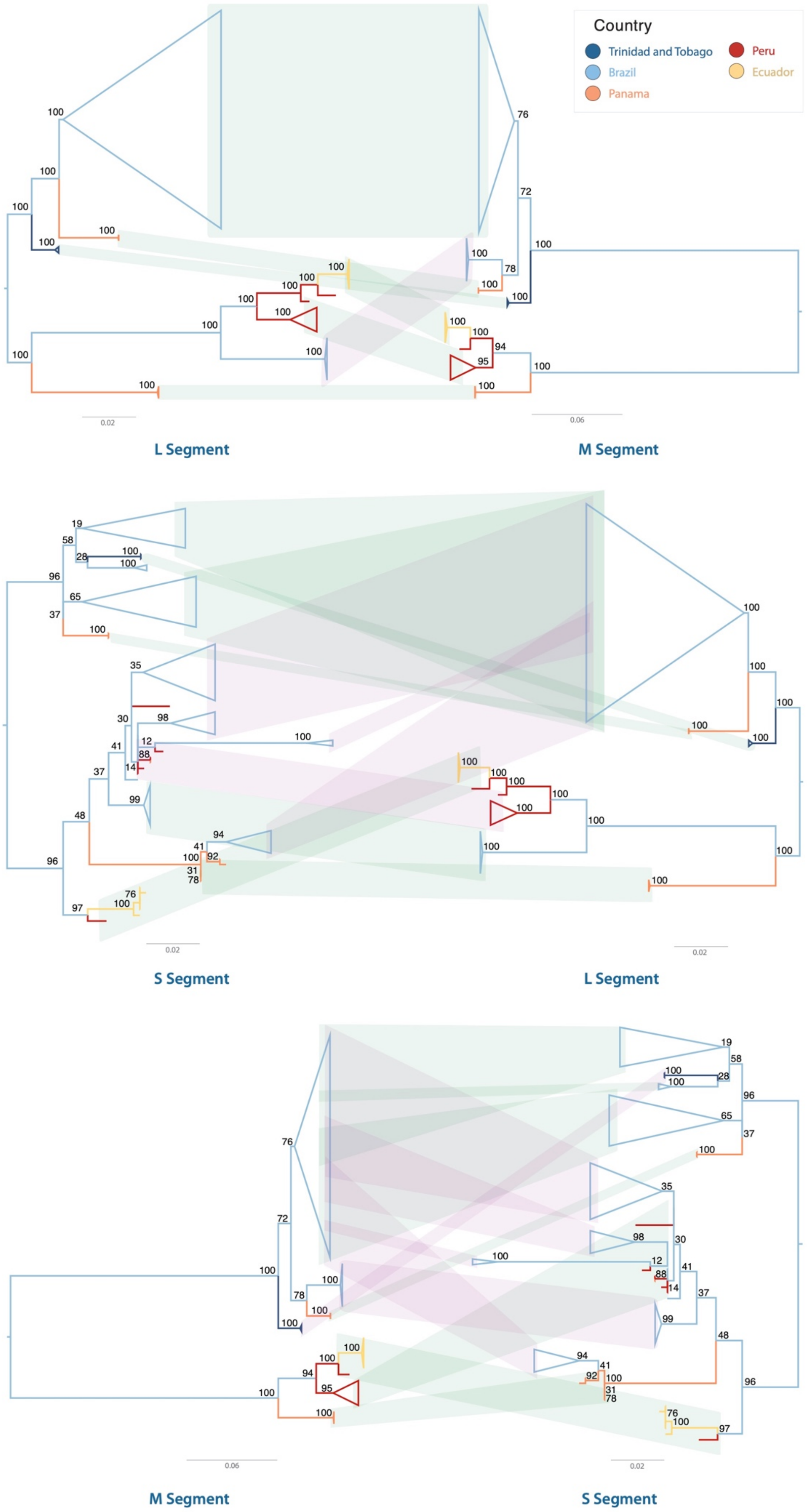
Country
Trinidad and Tobago
Brazil
Panama
Peru
Ecuador
0.02
0.06
L Segment
M Segment
0.02
0.02
S Segment
L Segment
0.06
0.02
M Segment
S Segment

Figure S3 (above). Pairwise comparison of maximum-likelihood phylogenetic trees of the three OROV genome segments suggest widespread reassortment. The L and S trees are midpoint rooted, and the M tree was re-rooted to maximise its topological congruency with its closest match, the L tree. Node support representing 100 bootstraps is shown on the tree nodes, where important monophyletic clades are collapsed in all trees (most collapsed nodes have high node support). All branches are coloured according to the most probable location using on a parsimony approach; colours match those used Fig. 1. Topologically similar clusters are highlighted in green, while potentially reassortant clusters are highlighted in purple.

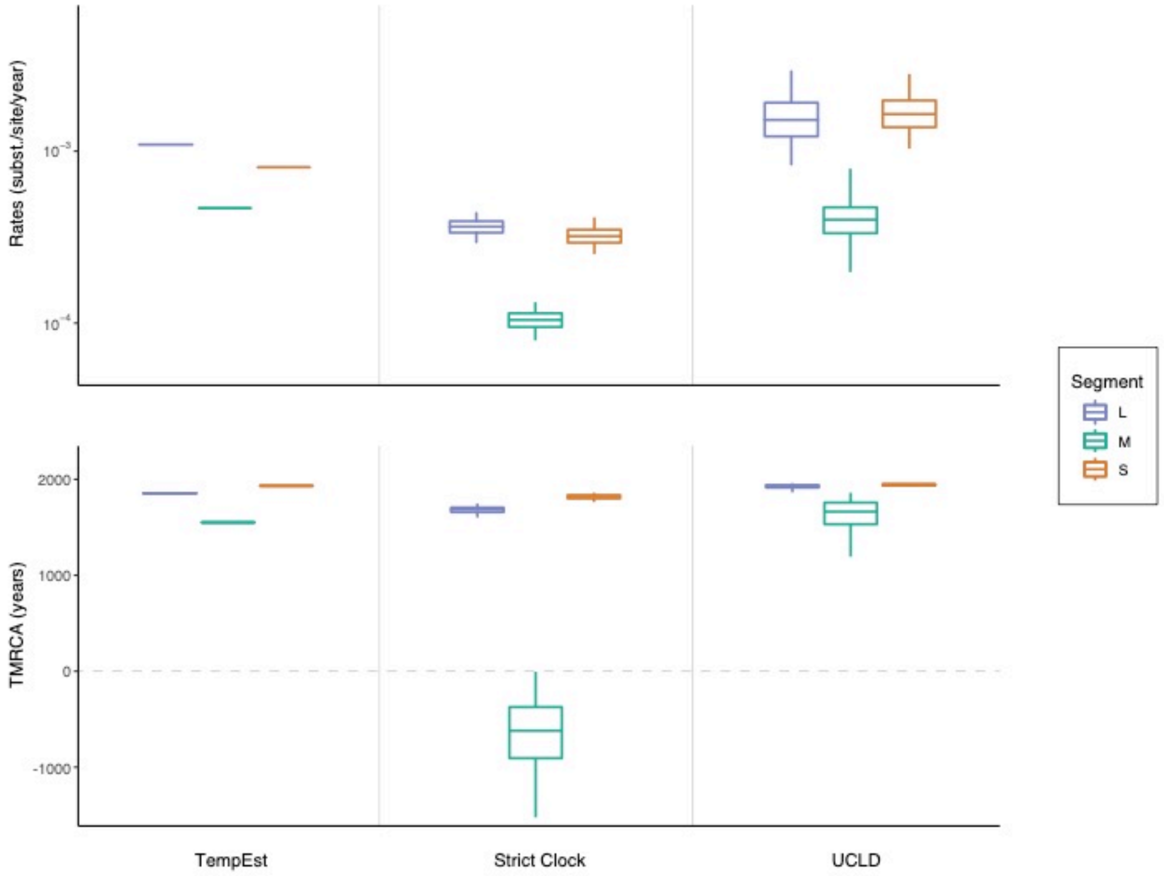

Figure S4 (above). Comparison of evolutionary rate estimate (upper panel) and times to the most recent common ancestor estimates (lower panel) for the three OROV genome segments estimated through different methods and clock models: a root-to-tip regression slope (TempEst), and a phylogenetically co-inferred rate using a strict clock model and a relaxed clock model (UCLD).

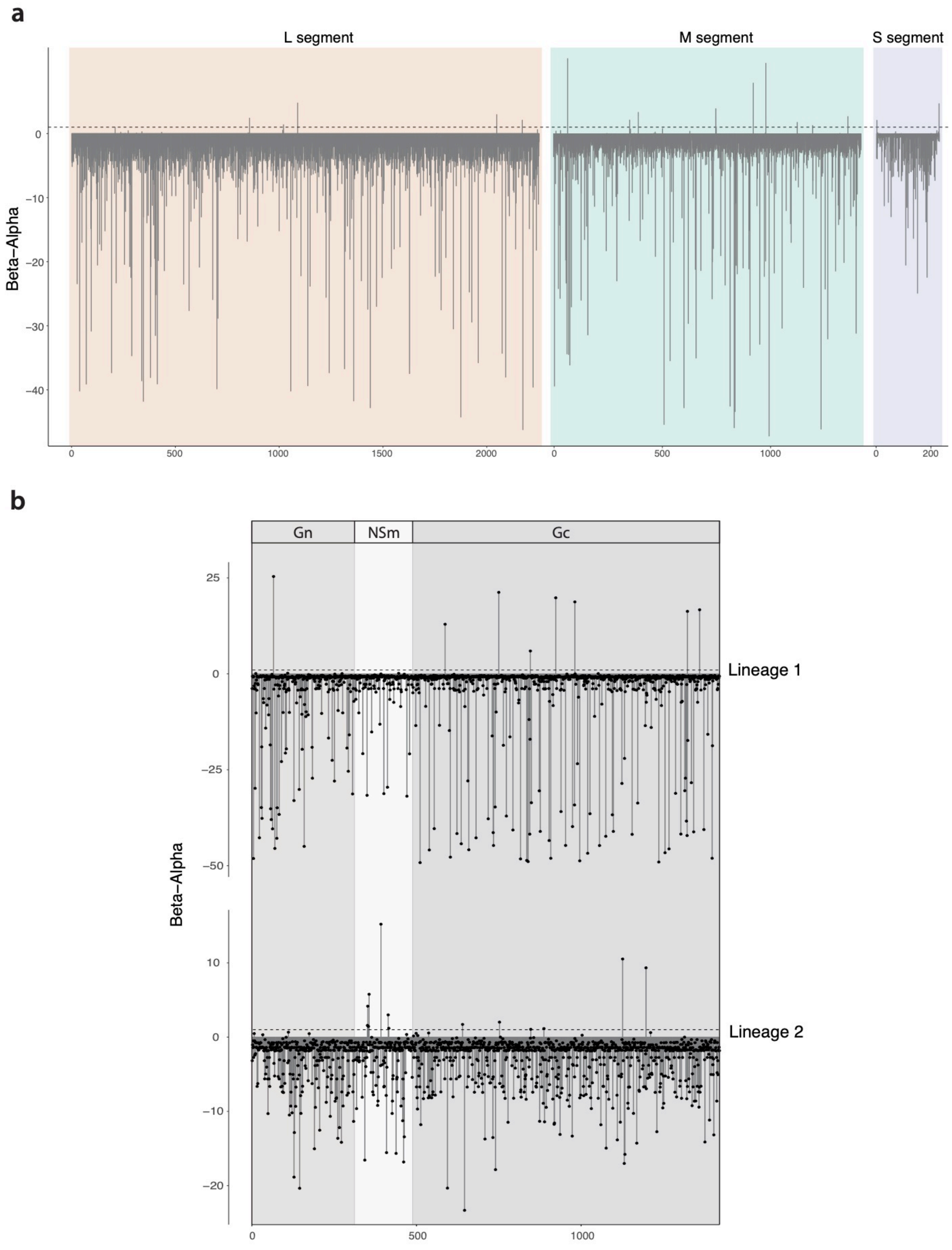

Figure S5. Site-wise differences between the non-synonymous (β) and synonymous (α) substitution rates in the OROV genome. The three genome segments (**a**) show a limited number of sites that evolve at a higher non-synonymous substitution rate (>1, marked by the

dotted line), suggestive of adaptive selection. Within the M segment (**b**), different codons (mostly on different protein reading frames) show evidence for adaptive selection.

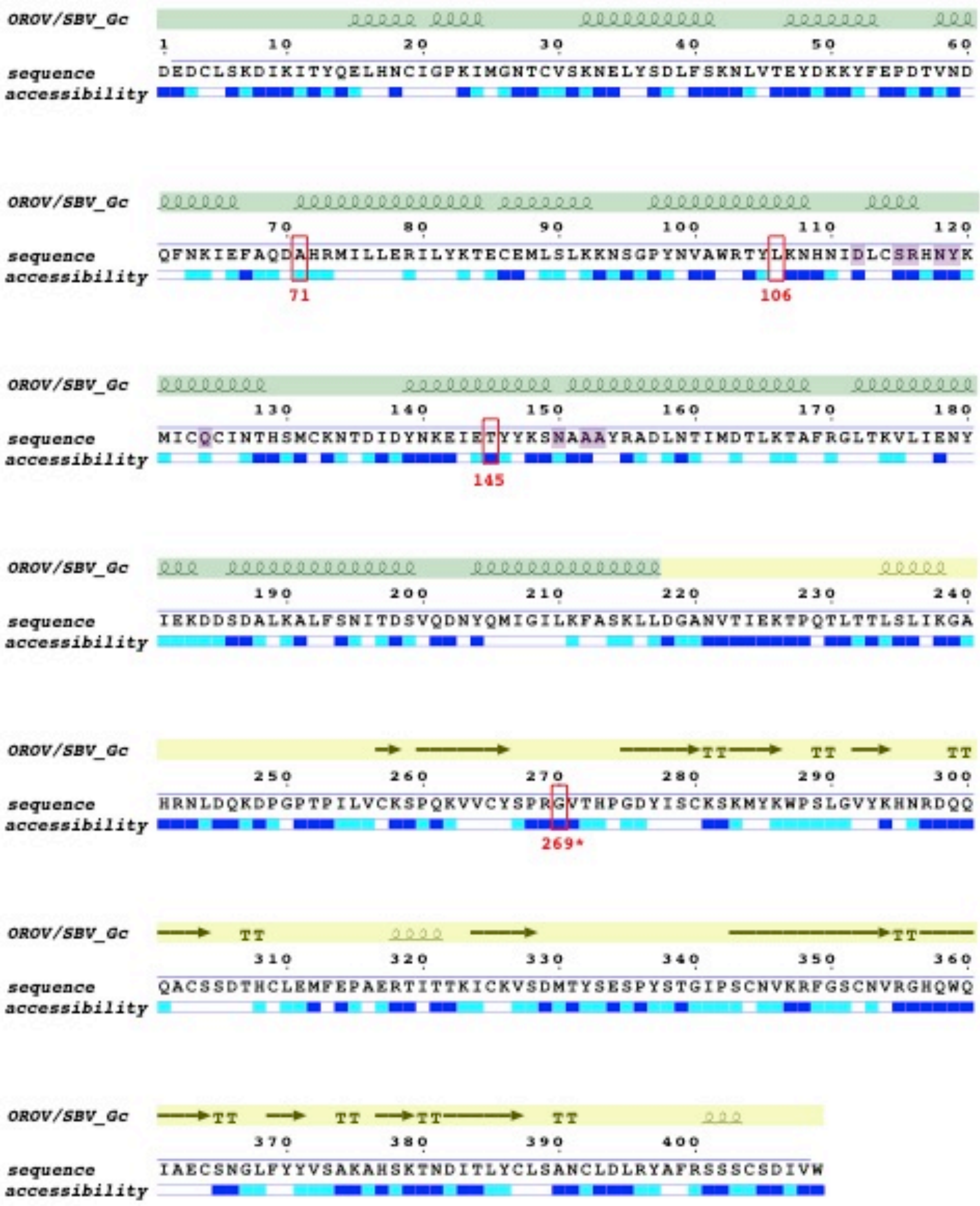

Figure S6 (above). Relative residue accessibility for the OROV/SBV composite model of the Gc protein, based on its atomic structure and amino acid sequence, using the ESPript server. Accessibility is scored as buried residues (white), intermediate residues (cyan) and exposed residues (blue). Red boxes show sites identified to be evolving under positive selection, with the red numbering highlighting the equivalent numbering at the OROV Gc sequence. An asterisk represents the site mapped onto the SBV component of the composite model, hence the numbering discrepancy. Secondary structures are shown above the sequence and highlighted based on the virus from which each component of the sequence was used to construct the composite model (green for OROV, yellow for SBV). Residues that directly play a role in the trimerisation interface of the head spike architecture, as identified in the PDBePISA server, are highlighted in purple.

Supplementary Tables

Table S1. Summary of all isolates included in this study, with accession numbers for each genome segment (including six new sequences from Ecuador).

ID	Host	City	Province/State	Country	Year	S	M	L
AC02	Homo sapiens	Xapuri	Acre	Brazil	1996	MG747503	MG747504	MG747505
AM01	Homo sapiens	Manaus	Amazonas (BR)	Brazil	1980	MG747506	MG747507	MG747508
AM03	Homo sapiens	Manaus	Amazonas (BR)	Brazil	1980	MG747509	MG747510	MG747511
MA02	Homo sapiens	Porto Franco	Maranhao	Brazil	1988	MG747512	MG747513	MG747514
MA03	Homo sapiens	Porto Franco	Maranhao	Brazil	1988	MG747515	MG747516	MG747517
MA06	Homo sapiens	Barra da Corda	Maranhao	Brazil	1993	MG747518	MG747519	MG747520
MG01	Callithrix sp.	Arino	Minas Gerais	Brazil	2000	MG747521	MG747522	MG747523
01-812-98	Homo sapiens	Iquitos	Loreto	Peru	1998	AF164553	---	---
D-057/057v	Homo sapiens	Esmeraldas	Esmeraldas	Ecuador	2016	MK506818	MK506823	MK506828
D-087/087	Homo sapiens	Esmeraldas	Esmeraldas	Ecuador	2016	MF926352	MF926353	MF926354
D-155/155v	Homo sapiens	Esmeraldas	Esmeraldas	Ecuador	2016	MK506819	MK506824	MK506829
D-171/171v	Homo sapiens	Esmeraldas	Esmeraldas	Ecuador	2016	MK506820	MK506825	MK506830
D-206/206v	Homo sapiens	Esmeraldas	Esmeraldas	Ecuador	2016	MK506821	MK506826	MK506831
D-210/210v	Homo sapiens	Esmeraldas	Esmeraldas	Ecuador	2016	MK506822	MK506827	MK506832
BeAn19991	Bradypus trydactylus	Sao Miguel do Guama	Para	Brazil	1960	KP052852	KP052851	KP052850
BeAn19991	Bradypus trydactylus	Sao Miguel do Guama	Para	Brazil	1960	AF164532	AF441119	KP052850
BeAn206119	Bradypus trydactylus	Maracana	Para	Brazil	1971	HQ830378	---	---
BeAn208402	Bradypus trydactylus	Maracana	Para	Brazil	1971	HQ830379	---	---
BeAn208819	Bradypus trydactylus	Maracana	Para	Brazil	1971	HQ830380	---	---
BeAn208823	Bradypus trydactylus	Maracana	Para	Brazil	1971	AY993912	---	---
BeAn626990	Callithrix sp.	Arino	Minas Gerais	Brazil	2000	AY117135	---	---
BeAr136921	Culex quinquefasciatus	Belem	Para	Brazil	1968	HM470111	---	---
BeAr366927	Culicoides paraensis	Belem	Para	Brazil	1979	HQ830448	---	---
BeAr473358	Culicoides paraensis	Porto Franco	Maranhao	Brazil	1988	AF164539	---	---
BeH244576	Homo sapiens	Belem	Para	Brazil	2009	HQ830444	---	---
BeH271078	Homo sapiens	Santarem	Para	Brazil	1975	HQ830445	---	---
BeH271815	Homo sapiens	Santarem	Para	Brazil	1975	AF164533	---	---
BeH29086	Homo sapiens	Belem	Para	Brazil	1961	HM470108	---	---
BeH355186	Homo sapiens	Tome-Acu	Para	Brazil	1978	HQ830446	---	---
BeH356898	Homo sapiens	Belem	Para	Brazil	1978	HQ830447	---	---
BeH366781	Homo sapiens	Belem	Para	Brazil	1979	HQ830449	---	---
BeH379693	Homo sapiens	Castanhal	Para	Brazil	1980	AF164534	---	---

Table S1. (Continued)

ID	Host	City	Province/State	Country	Year	S	M	L
BeH381114	Homo sapiens	Belem	Para	Brazil	1980	AF164535	---	---
BeH384192	Homo sapiens	Portel	Para	Brazil	1980	HQ830450	---	---
BeH384193	Homo sapiens	Portel	Para	Brazil	1980	HQ830451	---	---
BeH385591	Homo sapiens	Belem	Para	Brazil	1980	HQ830452	---	---
BeH389865	Homo sapiens	Manaus	Amazonas (BR)	Brazil	1980	HQ830453	---	---
BeH390233	Homo sapiens	Manaus	Amazonas (BR)	Brazil	1980	AF164536	---	---
BeH472200	Homo sapiens	Porto Franco	Maranhao	Brazil	1988	AF164537	---	---
BeH472204	Homo sapiens	Porto Franco	Maranhao	Brazil	1988	AF164538	---	---
BeH472433	Homo sapiens	Porto Franco	Maranhao	Brazil	1988	HQ830455	---	---
BeH472435	Homo sapiens	Porto Franco	Maranhao	Brazil	1988	HQ830456	---	---
BeH475248	Homo sapiens	Tucurui	Para	Brazil	1988	AF164540	---	---
BeH498913	Homo sapiens	Machadinho D'Oeste	Rondonia	Brazil	1990	HQ830457	---	---
BeH504514	Homo sapiens	Americano	Para	Brazil	1991	AF164541	---	---
BeH505442	Homo sapiens	Ouro Preto do Oeste	Rondonia	Brazil	1991	AF164542	---	---
BeH505663	Homo sapiens	Ariquemes	Rondonia	Brazil	1991	AF164543	---	---
BeH505764	Homo sapiens	Ariquemes	Rondonia	Brazil	1991	HQ830458	---	---
BeH505768	Homo sapiens	Ariquemes	Rondonia	Brazil	1991	HQ830459	---	---
BeH505805	Homo sapiens	Ariquemes	Rondonia	Brazil	1991	HQ830460	---	---
BeH521086	Homo sapiens	Barra da Corda	Maranhao	Brazil	1993	AY704559	---	---
BeH532314	Homo sapiens	Serra Pelada	Para	Brazil	1994	HQ830461	---	---
BeH532422	Homo sapiens	Serra Pelada	Para	Brazil	1994	HQ830462	---	---
BeH532490	Homo sapiens	Serra Pelada	Para	Brazil	1994	HQ830463	---	---
BeH532500	Homo sapiens	Serra Pelada	Para	Brazil	1994	HQ830464	---	---
BeH541863	Homo sapiens	Altamira	Para	Brazil	1996	AF164544	---	---
BeH543033	Homo sapiens	Oriximina	Para	Brazil	1996	AF164545	---	---
BeH543087	Homo sapiens	Xapuri	Acre	Brazil	1996	AF164547	---	---
BeH543091	Homo sapiens	Xapuri	Acre	Brazil	1996	HQ830465	---	---
BeH543100	Homo sapiens	Xapuri	Acre	Brazil	1996	HQ830466	---	---
BeH543618	Homo sapiens	Oriximina	Para	Brazil	1996	AF164548	---	---
BeH543639	Homo sapiens	Oriximina	Para	Brazil	1996	AY704562	---	---
BeH543733	Homo sapiens	Oriximina	Para	Brazil	1996	AY704560	---	---
BeH543760	Homo sapiens	Oriximina	Para	Brazil	1996	AY704568	---	---
BeH543857	Homo sapiens	Oriximina	Para	Brazil	1996	AY704566	---	---
BeH543880	Homo sapiens	Oriximina	Para	Brazil	1996	AY704565	---	---
BeH544552	Homo sapiens	Altamira	Para	Brazil	1996	AF164546	---	---
BeH622544	Homo sapiens	Parana	Tocantins	Brazil	2002	EF467368	---	---
BeH669314	Homo sapiens	Parauapebas	Para	Brazil	2003	EF467370	---	---
BeH669315	Homo sapiens	Parauapebas	Para	Brazil	2003	EF467369	---	---

Table S1. (Continued)

ID	Host	City	Province/State	Country	Year	S	M	L
BeH682426	Homo sapiens	Porto de Moz	Para	Brazil	2004	EF467371	---	---
BeH682431	Homo sapiens	Porto de Moz	Para	Brazil	2004	EF467372	---	---
BeH706890	Homo sapiens	Igarape Acu	Para	Brazil	2009	HQ830473	---	---
BeH706893	Homo sapiens	Igarape Acu	Para	Brazil	2009	HQ830474	---	---
BeH707157	Homo sapiens	Maracana	Para	Brazil	2006	HQ830476	---	---
BeH708139	Homo sapiens	Magalhaes Barata	Para	Brazil	2006	HQ830475	---	---
BeH708717	Homo sapiens	Maracana	Para	Brazil	2009	HQ830477	---	---
BeH758669	Homo sapiens	Mazagao	Amapa	Brazil	2009	HQ830479	---	---
BeH758687	Homo sapiens	Mazagao	Amapa	Brazil	2009	HQ830478	---	---
BeH759018	Homo sapiens	Mazagao	Amapa	Brazil	2009	HQ830486	---	---
BeH759021	Homo sapiens	Mazagao	Amapa	Brazil	2009	KP691608	KP691607	KP691606
BeH759022	Homo sapiens	Mazagao	Amapa	Brazil	2009	KP691611	KP691610	KP691609
BeH759023	Homo sapiens	Mazagao	Amapa	Brazil	2009	HQ830487	---	---
BeH759024	Homo sapiens	Mazagao	Amapa	Brazil	2009	KP691605	KP691604	KP691603
BeH759025	Homo sapiens	Mazagao	Amapa	Brazil	2009	KP691614	KP691613	KP691612
BeH759038	Homo sapiens	Mazagao	Amapa	Brazil	2009	HQ830484	---	---
BeH759040	Homo sapiens	Mazagao	Amapa	Brazil	2009	KP691617	KP691616	KP691615
BeH759041	Homo sapiens	Mazagao	Amapa	Brazil	2009	HQ830488	---	---
BeH759042	Homo sapiens	Mazagao	Amapa	Brazil	2009	HQ830489	---	---
BeH759043	Homo sapiens	Mazagao	Amapa	Brazil	2009	HQ830490	---	---
BeH759044	Homo sapiens	Mazagao	Amapa	Brazil	2009	HQ830491	---	---
BeH759146	Homo sapiens	Mazagao	Amapa	Brazil	2009	KP691632	---	---
BeH759483	Homo sapiens	Mazagao	Amapa	Brazil	2009	HQ830492	---	---
BeH759525	Homo sapiens	Mazagao	Amapa	Brazil	2009	HQ830480	---	---
BeH759529	Homo sapiens	Mazagao	Amapa	Brazil	2009	KP691620	KP691619	KP691618
BeH759531	Homo sapiens	Mazagao	Amapa	Brazil	2009	HQ830482	---	---
BeH759541	Homo sapiens	Mazagao	Amapa	Brazil	2009	HQ830481	---	---
BeH759558	Homo sapiens	Mazagao	Amapa	Brazil	2009	HQ830483	---	---
BeH759562	Homo sapiens	Mazagao	Amapa	Brazil	2009	HQ830485	---	---
BeH759620	Homo sapiens	Mazagao	Amapa	Brazil	2009	KP691623	KP691622	KP691621
DEI216	Homo sapiens	Iquitos	Loreto	Peru	1992	KP795074	KP795073	KP795072
GML444477	Homo sapiens	Chame	Panama	Panama	1989	AF164555	---	---
GML444479	NA	NA	Panama	Panama	1989	KC759130	KC759129	KC759128
GML444672	Homo sapiens	San Miguelito	Panama	Panama	1989	KP795077	KP795076	KP795075
GML444839	Homo sapiens	Bejuco	Panama	Panama	1989	KP795080	KP795079	KP795078
GML444911	Homo sapiens	Chame	Panama	Panama	1989	AF164556	---	---
GML445252	Homo sapiens	San Miguelito	Panama	Panama	1989	AF164557	---	---

Table S1. (Continued)

ID	Host	City	Province/State	Country	Year	S	M	L
GML450093	Homo sapiens	Chilibre	Panama	Panama	1989	AF164558	---	---
GML480914	Homo sapiens	NA	Panama	Panama	1989	KP795083	KP795082	KP795081
IQE7894	Homo sapiens	Iquitos	Loreto	Peru	2008	KP795086	KP795085	KP795084
IQT1690	Homo sapiens	Iquitos	Loreto	Peru	1992	KC759127	KP795088	KC759125
IQT1690*	Homo sapiens	Iquitos	Loreto	Peru	1995	KP795089	---	KP795087
IQT4083	Homo sapiens	Iquitos	Loreto	Peru	1997	KP795092	KP795091	KP795090
IQT4083	Homo sapiens	Iquitos	Loreto	Peru	1997	AF164552	---	---
IQT7085	Homo sapiens	Iquitos	Loreto	Peru	1998	KP795095	---	KP795093
IQT7085	Homo sapiens	Iquitos	Loreto	Peru	1998	AF164554	---	---
MD023	Homo sapiens	Madre de Dios	Madre de Dios	Peru	1993	AF164550	KP795097	KP795096
OBS9478	Homo sapiens	Bagua	Amazonas (PE)	Peru	2000	KP795101	KP795100	KP795099
PAN481126	Homo sapiens	Colon	Colon	Panama	1999	KP795104	KP795103	KP795102
TRVL9760	Homo sapiens	Vega de Oropouche	Trinidad	Trinidad	1955	KP026181	KP026180	KP026179
PA01	Ochlerotatus serratus	Ipixuna	Para	Brazil	1960	MG747524	MG7475215	MG747526
PA03	Homo sapiens	Belem	Para	Brazil	1961	MG747527	MG747528	MG747529
PA04	Homo sapiens	Belem	Para	Brazil	1961	MG747530	MG747531	MG747532
PA05	Homo sapiens	Braganca	Para	Brazil	1967	MG747533	MG747534	MG747535
PA06	Culex quinquefasciatus	Belem	Para	Brazil	1968	MG747536	MG747537	MG747538
PA07	Bradypus trydactylus	Maracana	Para	Brazil	1971	MG747539	MG747540	MG747541
PA08	Bradypus trydactylus	Maracana	Para	Brazil	1971	MG747542	MG747543	MG747544
PA09	Bradypus trydactylus	Maracana	Para	Brazil	1971	MG747545	MG747546	MG747547
PA14	Homo sapiens	Ananindeua	Para	Brazil	1978	MG747548	MG747549	MG747550
PA17	Culicoides paraensis	Belem	Para	Brazil	1979	MG747551	MG747552	MG747553
PA22	Homo sapiens	Belem	Para	Brazil	1980	MG747554	MG747555	MG747556
PA25	Homo sapiens	Serra Pelada	Para	Brazil	1994	MG747557	MG747558	MG747559
PA26	Homo sapiens	Serra Pelada	Para	Brazil	1994	MG747560	MG747561	MG747562
PA27	Homo sapiens	Serra Pelada	Para	Brazil	1994	MG747563	MG747564	MG747565
PA28	Homo sapiens	Serra Pelada	Para	Brazil	1994	MG747566	MG747567	MG747568
PA29	Homo sapiens	Altamira	Para	Brazil	1994	MG747569	MG747570	MG747571
PA34	Homo sapiens	Oriximina	Para	Brazil	1996	MG747572	MG747573	MG747574
PA38	Homo sapiens	Oriximina	Para	Brazil	1996	MG747575	MG747576	MG747577
PA39	Homo sapiens	Oriximina	Para	Brazil	1996	MG747578	MG747579	MG747580
PA41	Homo sapiens	Parauapebas	Para	Brazil	2003	MG747581	MG747582	MG747583
PA42	Homo sapiens	Parauapebas	Para	Brazil	2003	MG747584	MG747585	MG747586
PA43	Homo sapiens	Porto de Moz	Para	Brazil	2004	MG747587	MG747588	MG747589

Table S1. (Continued)

ID	Host	City	Province/State	Country	Year	S	M	L
PA43	Homo sapiens	Porto de Moz	Para	Brazil	2004	MG747587	MG747588	MG747589
PA44	Homo sapiens	Porto de Moz	Para	Brazil	2004	MG747590	MG747591	MG747592
PA47	Homo sapiens	Magalhaes Barata	Para	Brazil	2006	MG747593	MG747594	MG747595
PA49	Homo sapiens	Magalhaes Barata	Para	Brazil	2006	MG747596	MG747597	MG747598
PA50	Homo sapiens	Maracana	Para	Brazil	2006	MG747599	MG747600	MG747601
RO01	Homo sapiens	Machadinho D'Oeste	Rondonia	Brazil	1990	MG747602	MG747603	MG747604
RO05	Homo sapiens	Ariquemes	Rondonia	Brazil	1991	MG747605	MG747606	MG747607
Trinidad1961	Homo sapiens	Vega de Oropouche	Trinidad	Trinidad	1961	---	MF620128	MF620129

*June 1995 sequence excluded from analysis.

Table S2. Links to the data exploration tool Microreact for the three OROV genome segments analysed in this study.

OROV segment	Microreact link
L	https://microreact.org/project/OROV_America_L?lu=y
M	https://microreact.org/project/OROV_America_M?lu=y
S	https://microreact.org/project/OROV_America_S?lu=y

Table S3. Allele frequencies for codons under adaptive selection in the OROV genome.

Protein	Codon	Identity	Frequency
Gn	66	K	0.444
		R	0.333
		A	0.190
		T	0.032
NSm	86	R	0.651
		N	0.206
		S	0.143
Gc	269	D	0.656
		G	0.344
	442	T	0.734
		S	0.188
		I	0.078
